# Phylogenetic analysis of *Mycobacterium massiliense* strains having recombinant *rpoB* gene laterally transferred from *Mycobacterium abscessus*

**DOI:** 10.1371/journal.pone.0179237

**Published:** 2017-06-12

**Authors:** Byoung-Jun Kim, Ga-Na Kim, Bo-Ram Kim, Tae-Sun Shim, Yoon-Hoh Kook, Bum-Joon Kim

**Affiliations:** 1 Department of Microbiology and Immunology, Biomedical Sciences, Liver Research Institute and Cancer Research Institute, College of Medicine, Seoul National University, Seoul, Korea; 2 Division of Pulmonary and Critical Care Medicine, Department of Internal Medicine, University of Ulsan College of Medicine, Asan Medical Center, Seoul, Republic of Korea; Indian Institute of Technology Delhi, INDIA

## Abstract

Recent multi locus sequence typing (MLST) and genome based studies indicate that lateral gene transfer (LGT) events in the *rpoB* gene are prevalent between *Mycobacterium abscessus* complex strains. To check the prevalence of the *M*. *massiliense* strains subject to *rpoB* LGT (Rec-mas), we applied *rpoB* typing (711 bp) to 106 Korean strains of *M*. *massiliense* infection that had already been identified by *hsp65* sequence analysis (603 bp). The analysis indicated 6 smooth strains in *M*. *massiliense* Type I (10.0%, 6/60) genotypes but no strains in *M*. *massiliense* Type II genotypes (0%, 0/46), showing a discrepancy between the 2 typing methods. Further MLST analysis based on the partial sequencing of seven housekeeping genes, *argH*, *cya*, *glpK*, *gnd*, *murC*, *pta* and *purH*, as well as *erm*(41) PCR proved that these 6 Rec-mas strains consisted of two distinct genotypes belonging to *M*. *massiliense* and not *M*. *abscessus*. The complete *rpoB* sequencing analysis showed that these 6 Rec-mas strains have an identical hybrid *rpoB* gene, of which a 478 bp partial *rpoB* fragment may be laterally transferred from *M*. *abscessus*. Notably, five of the 6 Rec-mas strains showed complete identical sequences in a total of nine genes, including the seven MLST genes, *hsp65*, and *rpoB*, suggesting their clonal propagation in South Korea. In conclusion, we identified 6 *M*. *massiliense* smooth strains of 2 phylogenetically distinct genotypes with a specific hybrid *rpoB* gene laterally transferred from *M*. *abscessus* from Korean patients. Their clinical relevance and bacteriological traits remain to be elucidated.

## Introduction

Rapidly growing mycobacteria (RGM) are ubiquitous organisms that have gained increasing attention as important human pathogens. Recently, there have been more frequent reports regarding RGM pulmonary infections in many areas including South Korea [1, 2]. Within RGM, the *Mycobacterium abscessus* complex accounts for approximately 65–80% of RGM pulmonary infections [3]. In South Korea, the incidences of *M*. *abscessus* lung diseases have also been increasing and account for 70–80% of RGM-induced lung diseases in Korea [4–7]. *M*. *abscessus* can cause lung diseases in immunocompetent individuals and shares a number of characteristics with *M*. *tuberculosis*, including the ability to induce granulomatous lesions or caseous necrosis [8]. Together with *M*. *avium*, *M*. *abscessus* represents the most commonly isolated non-tuberculous mycobacteria (NTM) from cystic fibrosis (CF) patients. Infections with *M*. *abscessus* are difficult to treat, due to both natural broad-spectrum resistance and acquired resistance, with disparate antibiotic susceptibility patterns being observed between clinical strains [9].

The recent advanced taxonomic approach has revealed that the *M*. *abscessus* group could be divided into two subspecies, *M*. *abscessus* subsp. *abscessus* (the former species *Mycobacterium abscessus*) and *M*. *abscessus* subsp. *bolletii*. The *M*. *abscessus* subsp. *bolletii* was proposed to combine the two former species, *M*. *massiliense* and *M*. *bolletii* [10, 11]. It was recently reported that *M*. *massiliense* can be further subdivided into two genotypes (Type I and Type II) based on *hsp65* sequence analysis [12–14].

Hypothesis of lateral gene transfer (LGT) has been acknowledged as a major mechanism by which bacteria can acquire genetic diversity, for their survival under harsh environmental conditions [15, 16]. Although mycobacteria are assumed to be more recalcitrant to LGT compared to other bacteria, possibly due to the unusual cell wall structure and the relatively scarce exchange of genetic elements such as plasmids and transposable elements between strains within the genus [17, 18], there is increasing evidence that LGT plays an important role in the mycobacterial evolution from saprophytic organisms into opportunistic or specialized, highly persisting pathogens [19, 20], by the transfer of genes involved in niche change and antibiotic resistance [16, 21, 22]. Furthermore, recent multi locus sequence typing (MLST) and genome- based studies indicate that interspecies or intraspecies LGT events are prevalent in two *M*. *abscessus* subspecies, *M*. *abscessus* and *M*. *massiliense* [23]. These events are particularly more pronounced in the *rpoB* gene [23]. It showed that in subspecies differentiation using *rpoB* typing, the false identification rate was approximately 10% within the *M*. *abscessus* group, with the highest rate (up to 20%) in strains belonging to *M*. *massiliense*, suggesting a limitation of the *rpoB* typing method for identification within the *M*. *abscessus* group that has been most widely used for RGM differentiation [23].

In this study, we found some distinct *M*. *massiliense* strains with a hybrid *rpoB* gene, of which partial *rpoB* fragment may be laterally transferred from *M*. *abscessus*. Hereafter, we designate them Rec-mas strains. We sought to determine the molecular epidemiologic traits of Rec-mas strains in South Korea. To this end, we initially analyzed the prevalence of Rec-mas strains according to subspecies, genotype or morphotype within the *M*. *abscessus* group via MLST analysis and *erm*(41) PCR. We then analyzed the recombination profiles of the Rec-mas strains through a comparative analysis of the complete *rpoB* gene sequence.

## Methods and materials

### Mycobacterial strains and culture conditions

A total of 106 clinical strains of *M*. *massiliense* (60 Type I and 46 Type II strains) from different Korean chronic patients that had been identified and grouped into subspecies or genotype levels by *hsp65*- based methods [12] were used in this study (Table 1). *M*. *massiliense* Type I strains included both rough and smooth morphotypes (12 and 48 strains, respectively); however, Type II strains showed only rough morphotypes. All clinical strains were collected from the Asan Medical Center (Seoul, Republic of Korea) from January 2004 to June 2011. This work was approved by the institutional review board of Asan Medical Center (2012–0170), with documentation for waivers of informed consent. Each bacterial isolate was cultured for 3 days on Middlebrook 7H10 agar plates (supplemented with OADC), and colonies were sub-cultured in Middlebrook 7H9 broth media (supplemented with ADC) for 3 days at 37°C and 5% CO_2_ incubator. The bacteria were maintained as frozen stocks stored at -70°C by flash freezing of intermittent passaged samples with 20% glycerol. As type reference stains, *M*. *abscessus* ATCC 19977^T^ (= CIP 104536^T^), *M*. *abscessus* subsp. *bolletii* CIP 108541^T^ (hereafter referred to as *M*. *bolletii*) and *M*. *abscessus* subsp. *bolletii* CIP 108297 (= CCUG 48898) (hereafter referred to as *M*. *massiliense*) were also used in this study.

**Table 1:** Separation of 106 *M*. *massiliense* clinical strains into genotype level by sequence analyses based on the partial *hsp65* (603 bp) and *rpoB* (711 bp) gene sequences.

Genotype based on *hsp65*	No. (%)	*rpoB* based results (%)	
		*M*. *abscessus*	*M*. *massiliense*
Type I	60 (56.6)		
Rough	12 (5.8)	0 (0.0)	12 (11.3)
Smooth	48 (23.3)	6 (5.7)	42 (39.6)
Type II	46 (43.4)	0 (0.0)	46 (43.4)
Total	106 (100.0)	6 (5.7)	100 (94.3)

### DNA extraction, PCR and sequencing

Chromosomal DNA was extracted from each clinical isolate by the previously described bead beater- phenol extraction method [24] and then used as templates for PCR. The partial *hsp65* (644 bp) and *rpoB* (711 bp) gene targeted PCRs were applied to a total of 106 *M*. *massiliense* strains as described previously [12, 24]. To investigate the genetic diversities, MLST analyses targeting seven housekeeping genes were applied to six Rec-mas strains as previously described [25]. The seven target genes were *argH* (argininosuccinate lyase), *cya* (adenylate cyclase), *glpK* (glycerol kinase), *gnd* (6-phosphogluconate dehydrogenase), *murC* (UDP N-acetylmuramate-L-Ala ligase), *pta* (phosphate acetyltransferase), and *purH* (phosphoribosylaminoimidazole carboxylase ATPase subunit). This MLST scheme proved to be useful to study genetic diversity within *M*. *abscessus* complex strains [25, 26]. Because the *erm*(41)-targeted PCR was proposed as a simple method to differentiate *M*. *massiliense* from *M*. *abscessus* and *M*. *bolletii* species [27, 28], *erm*(41)-targeted PCR was applied to the reference and 6 Rec-mas strains. Additionally, to study the possibility of homologous recombination event among 6 Rec-mas strains, the complete *rpoB* gene sequence of each selected isolate was amplified by 5 primer sets; the detailed information for the primers is listed in S1 Table. All the primers used in this study are also listed in S1 Table. In the case of *erm*(41)-targeted PCR, the size of the amplified product was compared by visualization of the ethidium bromide (EtBr) stained electrophoresed agarose gel with a 1 kbp DNA marker. The template DNA (50 ng) and 20 pmol of each primer were added to a PCR premix tube (AccuPower PCR PreMix; Bioneer, Daejeon, South Korea). The final volume was adjusted to 20 μl with distilled water, and the PCR was conducted by subjecting the reaction mixtures to 5 min at 95°C, followed by 30 cycles of 95°C for 30 s, 60–65°C for 30 s, and 72°C for 1 min; a final extension at 72°C for 5 min was then performed using a model MyCycler^TM^ Thermal Cycler (Bio-Rad, Richmond, CA, USA). PCR products were purified using a MEGAquick-spin^TM^ Total Fragment DNA Purification Kit (iNtRON Biotechnology, Gyeonggi, South Korea) and then sequenced directly using each forward and reverse primer with an Applied Biosystems automated sequencer (model 377) and BigDye Terminator cycle sequencing kits (Perkin-Elmer Applied Biosystems, Warrington, United Kingdom). Both strands were sequenced as a cross-check. Gene sequences from the type strains of *M*. *abscessus* (CIP 104536^T^), *M*. *bolletii* (CIP 108541^T^) and *M*. *massiliense* (CIP 108297) were retrieved from the GenBank database.

### Sequence analyses

The sequences of two target genes [*hsp65* (603 bp) and *rpoB* (711 bp)] and 7 MLST target genes [*argH* (503 bp), *cya* (541 bp), *glpK* (563 bp), *gnd* (494 bp), *murC* (545 bp), *pta* (463 bp) and *purH* (549 bp)] were aligned with the ClustalW algorithm in the MEGA 4.0 program [29]. Phylogenetic trees based on each target gene or concatenated sequence were constructed by the neighbor-joining [30] and maximum parsimony [31] methods. All the phylogenetic analyses were evaluated in each case by a bootstrap analysis based on 1,000 replicates [32].

### Recombination analysis

To visualize the putative recombination site in the full *rpoB* gene sequence among 6 Rec-mas strains, the *rpoB* gene sequences of a *M*. *abscessus* Type strain (CIP 104536), a *M*. *massiliense* Type strain (CIP 108297), and two Rec-mas strains, Asan 50375 and 54142, were initially aligned by the MEGA 4.0 program and exported to the Recombination Detection Program (RDP4) software package for recombination analysis [33]. The recombination event and putative recombination break points were evaluated using the GENECONV, Chimaera, BootScan, MaxChi, and SiScan methods within the RDP4 program with default settings.

### Nucleotide sequence accession numbers

The determined *hsp65*, *rpoB* and 7 MLST gene sequences were deposited in GenBank under accession numbers from KX906854 to KX906903 and listed in S2 Table.

## Results

### Identification of *M*. *massiliense* strains with a recombinant *rpoB* gene (Rec-mas) by *rpoB* sequencing analysis

**Fig 1:**
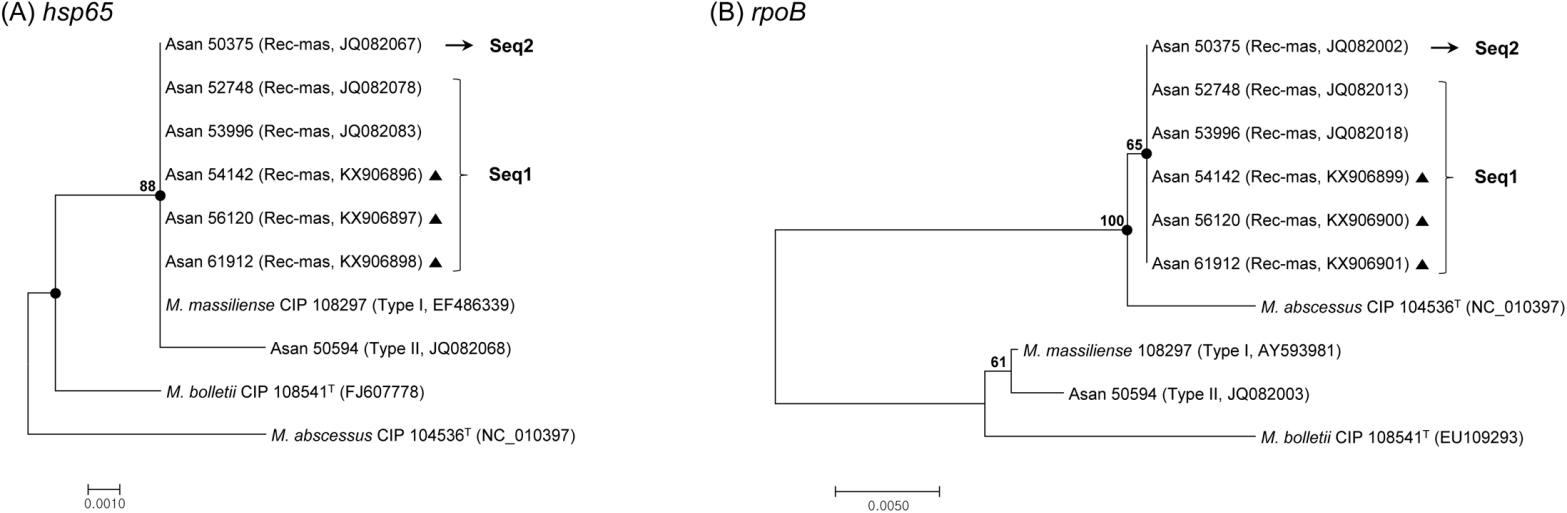
Neighbor-joining phylogenetic trees based on the partial *hsp65* and *rpoB* genes of 6 Rec-mass strains. Phylogenetic trees of 6 Rec-mas strains based on (A) the partial *hsp65* gene (603 bp) and (B) the partial *rpoB* gene (711 bp) sequences. All the trees were constructed using the neighbor-joining method in the MEGA 4.0 program. The bootstrap values were calculated from 1,000 replications and values <50% were not shown. Black-centered circles indicate that the corresponding clusters were supported with maximum parsimony-based trees. The bar indicates the number of base substitutions per site. Black-centered triangles indicate that the corresponding sequences were sequenced and obtained in this study.

The 711 bp *rpoB* sequence-based phylogenetic analysis indicated that 6 (5.7%, 6/106) and 100 strains (94.3%, 100/106) among 106 *M*. *abscessus* complex strains belonged to *M*. *abscessus* and *M*. *massiliense*, respectively, which had previously been identified as *M*. *massiliense* by 603 bp *hsp65* sequencing analysis (Table 1). The six strains showing discordant results with those of the *hsp65*-based analysis had identical *rpoB* sequences with each other, and only had a 5 bp mismatch (99.3% sequence homology) in 711-bp *rpoB* sequences with *M*. *abscessus* Type strain, CIP 104536^T^. All 6 of the potential *rpoB* recombinant strains of *M*. *massiliense* (Rec-mas) belonged to the *hsp65* Type I genotype with smooth colony morphologies (10.0%, 6/60). However, none of the rough or *hsp65* Type II strains had recombinant *rpoB* gene (Fig 1, Table 1).

### Phylogenetic analysis of 6 Rec-mas strains by single trees based on 7 MLST genes

**Fig 2:**
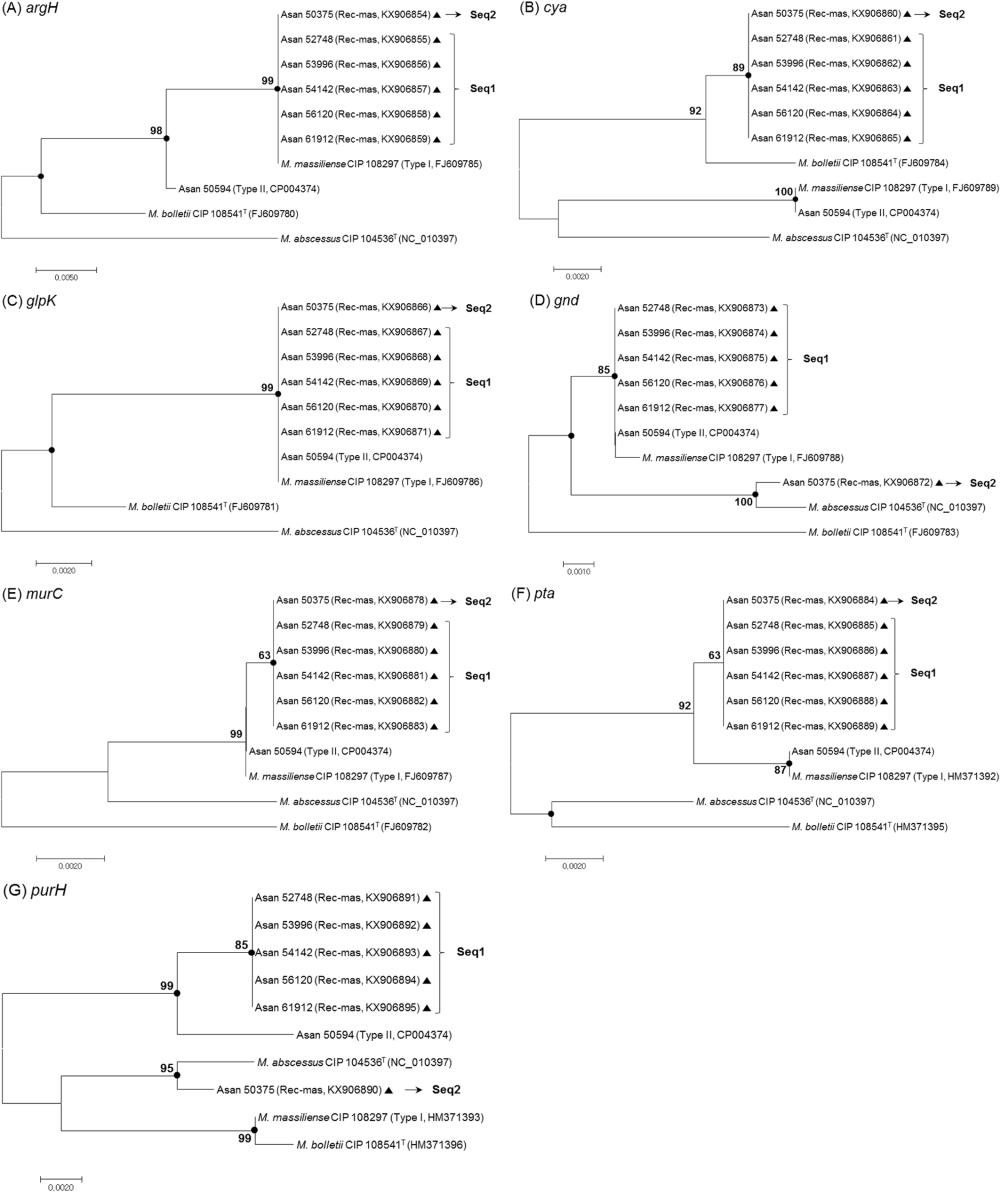
Neighbor-joining phylogenetic trees based on the 7 MLST genes of 6 Rec-mass strains. Phylogenetic trees of 6 Rec-mas strains from the partial sequencing of seven housekeeping genes. (A) *argH*, (B) *cya*, (C) *glpK*, (D) *gnd*, (E) *murC*, (F) *pta* and (G) *purH* gene sequence based trees were constructed by using the neighbor-joining method in the MEGA 4.0 program. The bootstrap values were calculated from 1,000 replications and values <50% were not shown. Black-centered circles indicate that the corresponding clusters were supported with maximum parsimony-based trees. The bar indicates the number of base substitutions per site. Black-centered triangles indicate that the corresponding sequences were sequenced and obtained in this study.

For the exact species delineation of the 6 Rec-mas strains, further MLST analyses based on the partial sequencing of seven housekeeping genes: *argH*, *cya*, *glpK*, *gnd*, *murC*, *pta* and *purH*, which had been previously applied to *M*. *abscessus* strains for the elucidation of recombination events [25], were also performed in this study. A single gene tree was built from the sequences for each of the seven genes in the MLST scheme for the separation of the 6 Rec-mas strains at the subspecies level of the *M*. *abscessus* complex (Fig 2). Our MLST data showed that the 6 Rec-mas strains consisted of 2 sequevars. The first sequevar (designated as Seq1) included 5 of the isolates, Asan 52748, Asan 53996, Asan 54142, Asan 56120 and Asan 61912, with identical sequences in a total of 9 genes, including 7 MLST genes, *hsp65* and *rpoB*. The other sequevar (designated as Seq2), isolate Asan 50375, had distinct MLST sequences. These two sequevars could be separated by 2 genes, *gnd* and *purH*, (Fig 2D and 2G) showing discordant clusters from each other. In the *gnd* gene sequence-based tree, 5 isolates of Seq1 closely clustered into *M*. *massiliense* CIP 108297^T^; however, Asan 50375 (Seq2) closely clustered into *M*. *abscessus* CIP 104536^T^ (Fig 2D). In the *purH* gene sequence-based tree, 5 isolates of Seq1 were closely clustered into *M*. *massiliense* Type II, Asan 50594; however, Asan 50375, Seq2, was closely clustered into *M*. *abscessus* CIP 104536^T^ (Fig 2G). The tree constructed from the *cya* gene showed that all 6 Rec-mas isolates clustered into *M*. *bolletii*, not into *M*. *massiliense* or *M*. *abscessus* (Fig 2B). In the *argH* and *pta* tree, all 6 Rec-mas isolates most closely clustered into *M*. *massiliense* CIP 108297^T^, showing identical sequences with each other (Fig 2A and 2F). In the *glpK* and *murC* trees, all 6 Rec-mas isolates clustered into *M*. *massiliense* CIP 108297^T^ and *M*. *massiliense* Type II genotype (Asan 50594), showing their close relationship with the *M*. *massiliense* group (Fig 2C and 2E). All the calculated sequence similarities of 9 genes (7 MLST genes, *hsp65* gene and *rpoB* gene) and their concatenated sequences between the reference strains of *M*. *abscessus* complex and 6 Rec-mas strains were shown in Table 2 and S3 Table.

**Table 2:** Comparison of 6 Rec-mas strains with reference strains of *M*. *abscessus* group in sequence similarities of 7 MLST, *hsp65* (603 bp) and *rpoB* (711 bp) gene sequences.

Genes (compared size, bp)	Sequence similarities between Seq1 (Seq2) and type or reference strains (%)		
	*M*. *abscessus* CIP 104536^T^	*M*. *massiliense* CIP 108297^T^	*M*. *massiliense* Type II strain (Asan 50594)
*argH* (503)	95.6 (95.6)	100.0 (100.0)	99.0 (99.0)
*cya* (541)	98.0 (98.0)	98.0 (98.0)	98.0 (98.0)
*glpK* (563)	98.0 (98.0)	100.0 (100.0)	100.0 (100.0)
*gnd* (494)	97.8 (99.4)	99.8 (97.8)	100.0 (98.0)
*murC* (545)	97.8 (97.8)	99.8 (99.8)	99.8 (99.8)
*pta* (463)	98.7 (98.7)	99.6 (99.6)	99.6 (99.6)
*purH* (549)	97.6 (99.5)	97.6 (98.4)	99.1 (97.6)
Concatenated 7 MLST genes (3,658)	97.6 (98.1)	99.2 (99.1)	99.3 (98.9)
*hsp65* (603)	98.8 (98.8)	100.0 (100.0)	99.7 (99.7)
*rpoB* (711)	99.3 (99.3)	97.0 (97.0)	96.8 (96.8)

### Phylogenetic analysis of 6 Rec-mas strains by trees based on concatenated sequences

**Fig 3:**
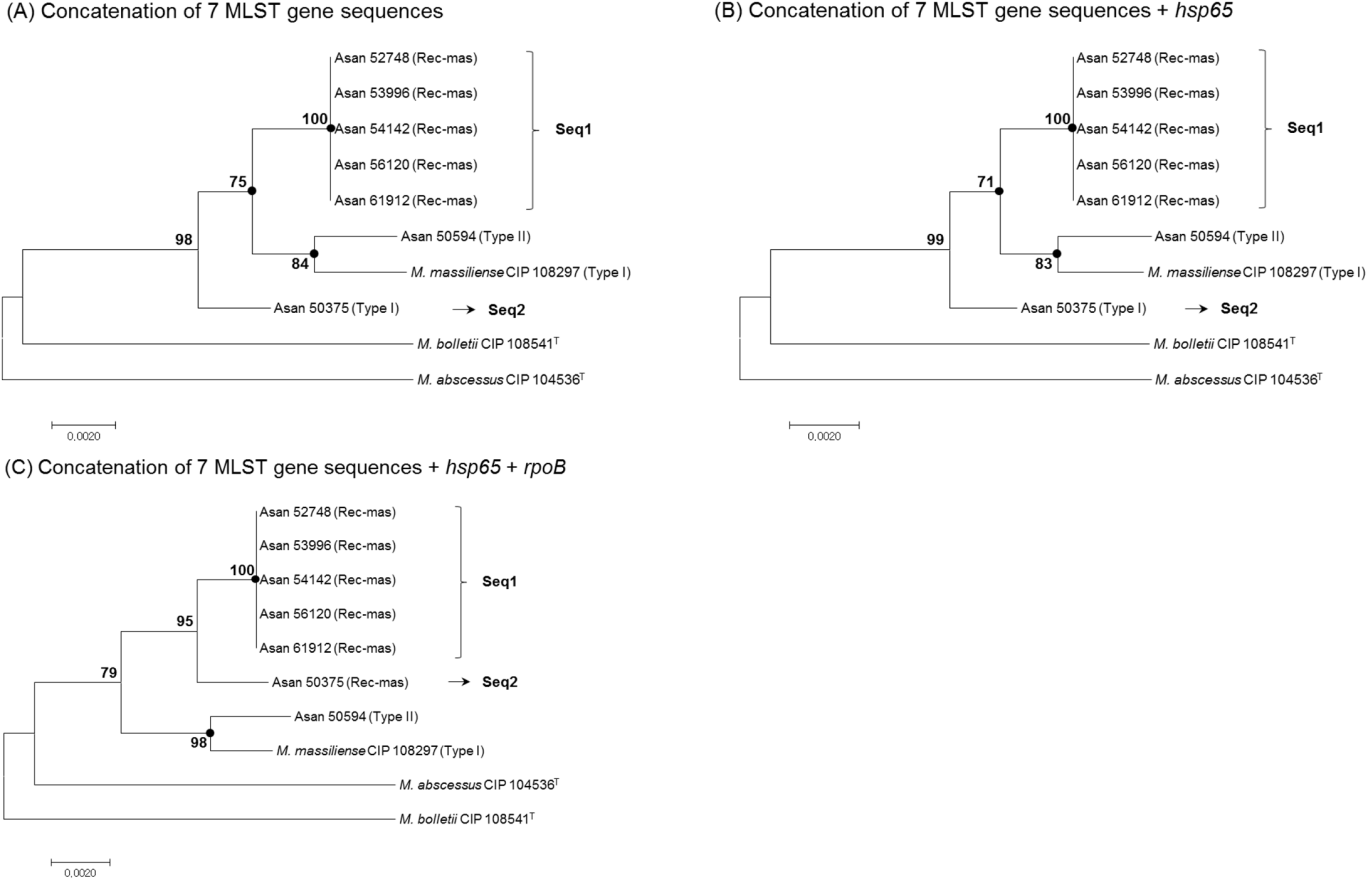
Neighbor-joining phylogenetic trees of 6 Rec-mas strains from concatenated sequences. The phylogenetic tree based on (A) concatenated 7 MLST gene sequences, (B) concatenated 7 MLST genes and *hsp65* gene sequences, and (C) concatenated 7 MLST genes, *hsp65* and *rpoB* gene sequences. The trees for all studied strains were generated by using the neighbor-joining method. Bootstrap support values (%) from 1,000 replications are indicated for each node and values <50% were not shown. Black centered circles indicate that the corresponding clusters were supported with maximum parsimony-based trees. The bar indicates the number of base substitutions per site.

First, the phylogenetic tree based on the concatenated sequences of the seven MLST genes showed that all 6 Rec-mas strains belonged to the *M*. *massiliense* group, including Type I and Type II, not to *M*. *abscessus* or *M*. *bolletii* (Fig 3A), as shown in the *hsp65* based analysis (Fig 1A), suggesting that the Rec-mas strains may be members of *M*. *massiliense* rather than *M*. *abscessus* and that their *rpoB* gene may be laterally gene transferred from *M*. *abscessus*. Second, in the *M*. *massiliense* cluster, Seq1 and Seq2 separately clustered while also being separated from the branch composed of Type I and II, suggesting that Seq1 and Seq2 may be two different novel genotypes in *M*. *massiliense* distinct from Type I or Type II.

Indeed, the sequence similarities between Seq1 and Seq2 (99.4%), between Seq1 and Type strain (Type I) (99.2%) or Type II (99.3%), or between Seq2 and Type strain (Type I) (99.1%) or Type II (98.9%) in concatenated sequences was the same as or lower than between Type strain (Type I) and Type II genotypes (99.5%), strongly supporting the above hypothesis (Table 2). Addition of the *hsp65* gene sequence into the 7 MLST gene concatenated sequences did not affect the entire topology of the tree obtained by the 7 MLST concatenated sequences, further strengthening our phylogenetic findings (Fig 3B). However, the addition of the *rpoB* gene sequence into the 7 MLST genes affected the phylogenetic location of Asan 50375 (Seq2 sequevar), possibly due to bias by LGT events in the *rpoB* gene of the Rec-mas isolates (Fig 3A and 3C).

### The separation of 6 Rec-mas strains by *erm*(41) PCR at the subspecies level

**Fig 4:**
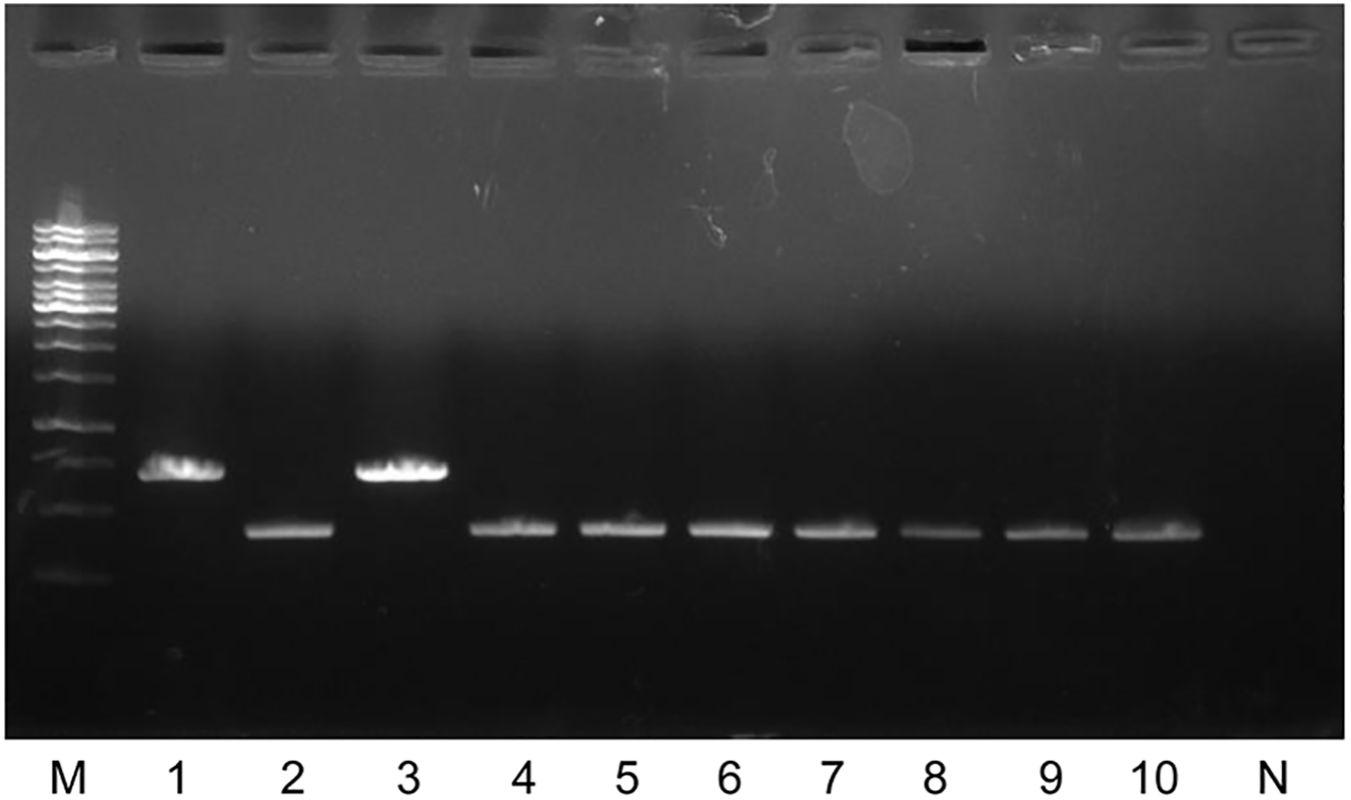
The identification of 6 Rec-mas strains into subspecies levels by PCR targeting the *erm*(41) gene. M, 1 kb DNA ladder; Lane 1, *M*. *abscessus* CIP 104536^T^; Lane 2, *M*. *massiliense* CIP 108297^T^; Lane 3, *M*. *bolletii* CIP 108541^T^; Lane 4, Asan 50594 (Type II); Lane 5, Asan 50375 (Type I, Seq2); Lane 6, Asan 52748 (Type I, Seq1); Lane 7, Asan 53996 (Type I, Seq1); Lane 8, Asan 54142 (Type I, Seq1); Lane 9, Asan 56120 (Type I, Seq1); Lane 10, Asan 61912 (Type I, Seq1); N, negative control.

The *M*. *massiliense erm*(41) gene is reported to have a large C-terminal deletion. Therefore, the *erm*(41) PCR could be used as a simple method to differentiate *M*. *massiliense* from *M*. *abscessus* and *M*. *bolletii* species [27, 28]. To further confirm the authenticity of the 6 Rec-mas strains, we applied *erm*(41) PCR to them, showing that unlike *M*. *abscessus* and *M*. *bolletii* Type strains producing a full-size product (approximately 700 bp), the 6 Rec-mas strains produced a shorter product (approximately 350 bp) identical with the *M*. *massiliense* Type strain (Fig 4).

### Recombination analysis of Rec-mas strains using the complete *rpoB* sequence

**Fig 5:**
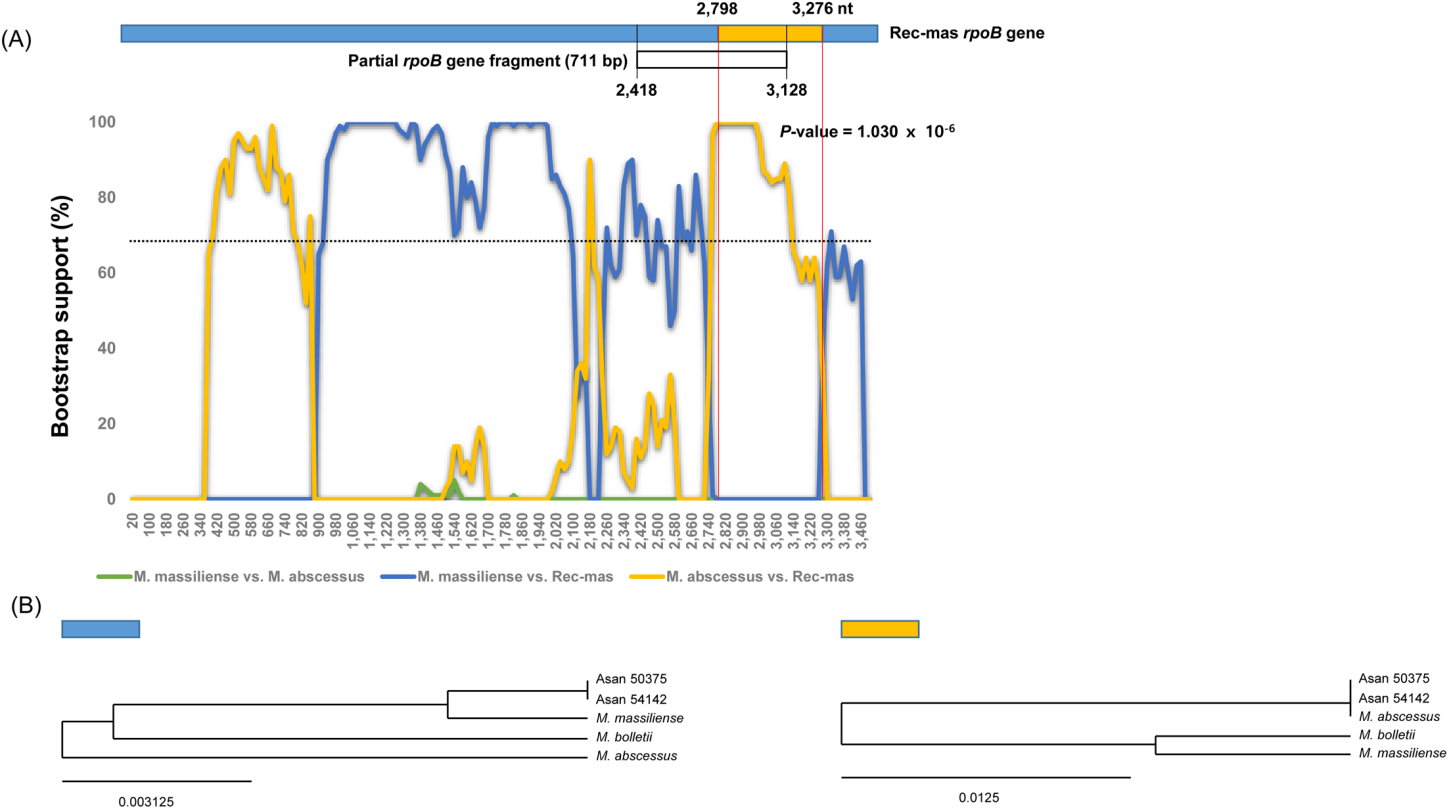
Recombination analysis of Rec-mas strains in the complete *rpoB* gene sequence. (A) A BootScan Plot was constructed based on a pairwise distance model in the RDP4 program. A BootScan support percent of over 70% (cutoff value, dotted line) was considered significant. The green line represents the comparison of *rpoB* sequences from *M*. *abscessus* and *M*. *massiliense* Type strains. The blue line represents the comparison of *rpoB* sequences from *M*. *massiliense* Type and Rec-mas (Asan 50375 and 54142) strains. The yellow line represents the comparison of *rpoB* sequences from *M*. *abscessus* Type and Rec-mas (Asan 50375 and 54142) strains. The estimated breakpoints are tagged on the schematic diagram of the Rec-mas *rpoB* gene. (B) Neighbor-joining trees based on the *rpoB* sequences excluding the 478 bp recombination region (blue) and only the recombination region (yellow).

To analyze the recombination events in the *rpoB* gene sequence of the 6 Rec-mas strains, we sequenced their complete *rpoB* genes. All 6 Rec-mas strains had identical complete *rpoB* sequences, irrespective of the sequevar type, as shown in the partial 711 bp *rpoB* sequences. The entire *rpoB* sequence based phylogenetic tree also showed that they all belonged to *M*. *abscessus*, not to the *M*. *massiliense* group (Type I or Type II genotypes), as shown in the 711 bp *rpoB*-based tree (data not shown). To confirm the possibility of recombination events in their *rpoB* gene sequence from *M*. *abscessus* to Rec-mas strains, aligned complete *rpoB* gene sequences were analyzed using the RDP4 program. Fig 5A depicts the results of the BootScan analysis of the Rec-mas strains, which revealed that the locations of the possible recombination breakpoints were estimated at 2,798 and 3,276 nt with statistical significance (*p* value: 1.030 x 10^−6^), which contains the 711 bp *rpoB* fragment, a major target for mycobacterial identification [34]. A phylogenetic tree based on the *rpoB* sequences but excluding the 478 bp recombination region showed that, in contrast to trees based on the recombination region (478 bp) of *rpoB* sequences, the phylogenetic location of the Rec-mas strain was changed to *M*. *massiliense*, strongly supporting the above findings (Fig 5B).

## Discussion

Recent genomic studies targeting *M*. *abscessus* complex strains showed that they have acquired large genetic diversity by LGT from non-mycobacterial species sharing a similar ecological niche or by homologous recombination both within and between subspecies, which may contribute to different epidemiological traits, distinct virulence and different clinical outcomes [35–38]. Several MLST schemes for the separation of members of the *M*. *abscessus* complex were developed and have been applied to a large number of clinical strains from different geographical sources [23, 25, 26, 39, 40]. The MLST studies also showed that some *M*. *abscessus* strains have a composite genetic pattern including housekeeping genes from different subspecies, suggesting the frequent occurrence of homologous recombination between subspecies of *M*. *abscessus* [7, 23, 41, 42].

Among developed MLST schemes, the MLST scheme using seven housekeeping genes showed relatively low allelic diversity (among *M*. *abscessus*: 0.60–3.46%; *M*. *massiliense*: 0.41–4.99%) [39], but this proved to be sufficient for identifying diverse sequence types (STs) within the *M*. *abscessus* complex based on previous reports [23, 25, 39, 40]. Both the *hsp65* and *rpoB* genes have been widely used in the identification of *M*. *abscessus* complex strains [43, 44]. Therefore, in this study, we used phylogenetic analysis based on sequences of a total of nine genes, including *hsp65*, *rpoB* and the seven MLST genes for the genotypic characterization of the 6 Rec-mas strains.

There are several worthwhile findings in this study. First, a comparison of the results obtained by *hsp65*- and *rpoB*-based analysis in 106 Korean pulmonary patients revealed a total of 6 Rec-mas strains (5.7%) in which the *rpoB* gene was laterally transferred from *M*. *abscessus* (Table 1 and Fig 1). Our MLST gene evaluation and *erm*(41) PCR analysis clearly showed that these strains are members of *M*. *massiliense* (Figs 2–4 and Table 2) and not *M*. *abscessus*, suggesting that *hsp65* analysis may be more reliable for the identification of *M*. *abscessus* complex strains at the subspecies level, compared to the *rpoB*-based method.

Second, our data also demonstrated that these 6 Rec-mas strains consisted of two novel distinct genotypes, Seq1, including 5 isolates (Asan 52748, 53996, 54142, 56120 and 61912), and Seq2, including only one isolate, Asan 50375, and were phylogenetically separated from the reported two *hsp65* genotypes of *M*. *massiliense*, Type I and Type II. Seq1 and Seq2 strains identified in this study were also distinct from several STs shown in the MLST database of the *M*. *abscessus* complex based on seven housekeeping genes (https://pubmlst.org/mabscessus/) [39]. Although Seq1 strains are almost identical to ST7 strains, they have a distinct *pta* sequence. In the case of the Seq2 strain, the *gnd* and *purH* genes were clustered into *M*. *abscessus*, but not *M*. *massiliense*, in the phylogenetic trees based on each gene (Fig 2D and 2G), suggesting the possibility of LGT, not only in the *rpoB* gene, but also in the *gnd* and *purH* genes in this genotype. This result further highlights the importance of multi-gene-based analysis for the identification of *M*. *abscessus* complex strains. In particular, the tree based on the concatenated sequences of 7 MLST genes, as well as each single gene-based tree, showed that the five isolates of Seq1 have completely identical sequences (Figs 2 and 3) despite being from different pulmonary patients, suggesting their clonal propagation among Korean patients. Identical sequences between the five Seq1 isolates in the complete *rpoB* and the partial *hsp65* sequences were also found, which strongly supports the above hypothesis.

Third, all 6 Rec-mas strains showed smooth colony morphotypes and *hsp65* sequences identical to those of Type I, but not Type II, which always showed rough colony morphotypes due to the large deletion of GPL in their genomes [14]. Several reports have found a correlation between rough colony morphology and virulence including invasive infection [45–47]. Thus, Type II strains could be regarded as being more adapted to intracellular parasitism than Type I strains. Considering our finding that the Rec-mas strains are phylogenetically closer to the *M*. *massiliense* reference strain CIP 108297 (Type I) than the Type II prototype, Asan 50594 (Table 2 and Fig 3), it is tempting to speculate that the *rpoB* gene of the Rec-mas strains may have been laterally transferred from *M*. *abscessus* at the evolutionary stage of free living in soil or aquatic environments rather than during the intracellular parasitism stage.

Fourth, for the first time, we determined the putative recombination sites of the Rec-mas strains in their complete *rpoB* sequences via BootScan analysis using the RDP4 program [33] (Fig 5). We found that the putative recombination fragment of approximately 478 bp overlapped within the C-terminal region of the partial 711 bp *rpoB* fragment, a major target for mycobacterial identification, providing a likely explanation for the misidentification of Rec-mas strains by this method. This finding also provides information that should be used for the design of primers or probes to avoid the misidentification of the *M*. *abscessus* complex or the selective detection of Rec-mas strains.

Finally, a limitation of this study is not having whole genome sequences for comparison. To decide the exact taxonomic location of the 6 Rec-mas strains within the *M*. *abscessus* group, comparative genome analysis using their whole genome sequences should be performed in the future.

In conclusion, we have identified 6 *M*. *massiliense* smooth strains of a phylogenetically distinct genotype, with a specific hybrid *rpoB* gene laterally transferred from *M*. *abscessus*, in Korean patients. Our data suggest that LGT may be one of the driving forces that contribute to the diversity or evolution of *M*. *abscessus* strains. Furthermore, it also highlights the importance of multi-gene-based analysis in minimizing the risk of mis-identification within *the M*. *abscessus* complex strains. Their clinical relevance and bacteriological traits remain to be elucidated.

## Supporting information

S1 Table Primer sets used for PCR ampilification and sequencing in this study. (XLSX)

S2 Table GenBank accession numbers corresponding to obtained sequences in this study. (XLSX)

S3 Table Sequence similarities of *hsp65*, *rpoB*, 7 MLST genes and concatenated sequences among *M. abscessus* complex strains. (XLSX)
